# Ecological momentary assessment of daily patient-reported outcomes and actigraphy-measured physical activity and sleep in patients with rheumatoid arthritis and spondyloarthritis: a study protocol

**DOI:** 10.1136/bmjopen-2025-113370

**Published:** 2026-02-10

**Authors:** Nathan Aymard, Agathe Darmaillacq, Sebastien Bailly, Amélie Kechichian, Sébastien Baillieul, Chloé Bernardy, Romain Gastaldi, Patrice Flore, Athan Baillet, Monique Mendelson

**Affiliations:** 1 Univ. Grenoble Alpes, HP2 laboratory, Inserm U1300, Grenoble, France; 2 Univ. Grenoble Alpes, CNRS, UMR 5525, TIMC, VetAgro Sup, Grenoble INP, Grenoble, France; 3 Neurorehabilitation Department, Institute of Rehabilitation, Grenoble Alpes University Hospital, Grenoble, France; 4 Univ. Grenoble-Alpes, SENS Laboratory, Grenoble, France; 5 Univ. Grenoble Alpes, Department of Rheumatology, CHU Grenoble Alpes, Grenoble, France

**Keywords:** RHEUMATOLOGY, Fatigue, Mobile Applications

## Abstract

**Background:**

Rheumatoid arthritis (RA) and spondyloarthritis (SpA) are chronic inflammatory rheumatic diseases characterised by pain, fatigue, mood disturbances, sleep problems and reduced quality of life. These symptoms are highly variable both between individuals and within individuals across days, reflecting the fluctuating nature of disease activity and daily functioning. Although physical activity is known to alleviate many of these symptoms, individuals with RA and SpA often encounter barriers that limit regular engagement. Capturing the dynamic interplay between symptoms and physical activity therefore requires methods that account for day-to-day and moment-to-moment variability. Ecological momentary assessment (EMA), especially when combined with actigraphy, enables real-time, context-sensitive monitoring of symptoms and physical activity in daily life. However, little is known about the feasibility and acceptability of such protocols in individuals with RA and SpA, for whom participant burden and adherence may represent significant challenges. This pilot study therefore aims to assess the feasibility and acceptability of a 14-day EMA protocol and to explore factors associated with objectively measured physical activity in individuals with RA and SpA.

**Methods and analysis:**

50 adults diagnosed with RA or SpA will be recruited through rheumatology clinics or via advertisement. Eligible participants must be smartphone users without cognitive or physical impairments affecting participation. After providing consent, participants will complete baseline questionnaires regarding disease activity, quality of life, sleep, pain, fatigue, affective states and will attend a remote session with a member of the research team to learn how to use the mobile app. They will then complete a 14-day EMA protocol, during which data on patient-related outcomes (PROs), including pain, fatigue, sleep quality and affective states (i.e. positive and negative affects) will be assessed four times daily: upon awakening, 11:00, 15:00 and 20:30. Physical activity and sleep will be continuously monitored using both a wrist-worn and a thigh-worn device. Feasibility will be evaluated based on adherence to EMA prompts and actigraphy wear time. Acceptability will be assessed via a study-specific questionnaire and qualitative interviews conducted at the end of the protocol. Exploratory analyses will examine real-time, temporal and lagged relationships between PROs (pain, fatigue affective states), sleep and physical activity levels.

**Ethics and dissemination:**

This study was approved by the French national ethics committee [*Comité de protection des personnes Nord Ouest I, 2025-A01349-40*] on 24/07/2025. The results will be disseminated in peer-reviewed journals and at international conferences.

**Trial registration number:**

NCT07167784.

**STRENGTHS AND LIMITATIONS OF THIS STUDY**

- This study uses concurrent ecological momentary assessment and wrist-worn and thigh-worn actigraphy to capture sleep and physical activity in real-world conditions.
- A 14-day intensive repeated-measures design enables within-person analysis using both subjective and objective measurements.
- The pilot feasibility design and limited sample size restrict the scope of analyses and generalisability of findings.
- The protocol was co-developed with a patient research partner to optimise the clarity and feasibility of EMA items.
- The remote, technology-based design with frequent assessments may increase participant burden and limit inclusion of individuals with low digital literacy.

## Introduction

 Patient-reported outcome measures (PROs), including pain, fatigue, mood and sleep quality, are major determinants of reduced quality of life in individuals with rheumatoid arthritis (RA) and spondyloarthritis (SpA).^1 2^ These chronic conditions affect the joints and surrounding tissues and are characterised by symptoms that fluctuate markedly within and between days.^3^,^6^ Pain, fatigue and sleep disturbances frequently co-occur and interact dynamically, influencing patients’ daily functioning and levels of physical activity.^7 8^ As a result, the burden of RA and SpA is not only defined by average symptom levels but also by their day-to-day variability and temporal interactions, which are insufficiently captured by traditional cross-sectional or clinic-based assessments.

In addition to pharmacological treatments, such as nonsteroidal anti-inflammatory drugs and conventional or biological disease-modifying antirheumatic drugs, regular physical activity (PA) and reduced sedentary behaviour are strongly recommended for patients with RA and SpA.^9^,^11^ Current European League Against Rheumatism guidelines emphasise regular PA as a core component of disease management due to its beneficial effects on pain, physical function, mobility and disease activity.^12^,^16^ However, despite these established benefits, many patients struggle to maintain regular PA in daily life, with pain, fatigue and sleep disturbances commonly reported as major barriers.^17^,^19^ Understanding how fluctuations in symptoms relate to everyday activity patterns in real-world settings is therefore essential to identify periods of vulnerability and opportunity and to inform more personalised and adaptive PA interventions.

Most existing studies have relied on retrospective symptom assessments (eg, questionnaires), which fail to capture the day-to-day variability in PROs progression and PA levels. Ecological Momentary Assessment (EMA), which involves repeated real-time data collection via mobile applications, reduces recall bias and enhances the ecological validity of data, particularly those related to PROs.^20^ When combined with actigraphy, EMA enables a fine-grained understanding of the dynamic interactions between PROs and PA in daily life. Although this combined approach has been applied in other chronic diseases,^21 22^ it has not yet been explored in patients with RA and SpA.

Although EMA provides numerous advantages, its implementation also presents challenges.^23^ Maintaining participant adherence and managing the burden of frequent prompts can lead to response fatigue and habituation effects.^24^ Suboptimal adherence to the EMA protocol not only generates missing data but may also compromise study outcomes. A meta-analysis of 68 EMA studies on health-related behaviours in adults reported an average adherence rate of 82%, with considerable variability ranging from 38% to 98%.^25^ This variability highlights the importance of evaluating feasibility and adherence within specific clinical populations and study designs before large-scale implementation. In this context, feasibility refers to the extent to which study procedures can be successfully implemented in real-world conditions with the target population, typically assessed through recruitment and retention rates, adherence to repeated assessments and participant acceptability. Given this variability, it is essential to evaluate the feasibility and acceptability of EMA protocols in specific populations before large-scale implementation. This pilot study aims to assess the feasibility and acceptability of a 14-day EMA protocol combined with actigraphy in patients with RA and SpA. The secondary objectives of this study are: (1) to examine the temporal relationships between pain, fatigue and sleep, considering their reciprocal influences and variations across different times of the day; (2) to describe both the immediate and lagged effects of these factors on objectively measured physical activity; (3) to compare adherence to and acceptability of two actigraph devices assessing physical activity, sleep and sedentary behaviour, worn at two different body sites (non-dominant wrist vs thigh) and (4) to explore the qualitative determinants of protocol acceptability directly from the patients’ perspective.

## Materials and methods

### Study design and setting

The **D**aily s**Y**mptom a**N**d phys**I**c**A**l activity **M**onitoring In **C**hronic inflammatory rheumatic diseases (DYNAMIC) study is a prospective, observational, 14-day study conducted entirely remotely. Data will be collected using actigraphy and repeated questionnaires delivered via a mobile application.

Participants will wear two actigraphy devices (Actigraph GT3X, Actigraph, Pensacola, FL, USA and SENS motion, SENS, Copenhagen, Denmark) continuously for 14 days, while completing EMA surveys four times per day via the Avicenna Research App (Avicenna Research, Toronto, Canada).

Upon providing consent, participants will be enrolled in the study. Actigraphy devices will be mailed to them, and a meeting will be scheduled upon receipt. During this session, participants will complete baseline questionnaires online.

Participants will then be instructed to continuously wear two actigraphy devices (Actigraph GT3x and SENS motion) and to complete EMA surveys via the Avicenna Research app (Avicenna Research, Toronto, Canada). To optimise engagement and adherence, the EMA protocol will incorporate a gamification component specifically designed to increase response rates.^26^ At study initiation, participants will complete a conditional item assessing their usual wake-up time (selectable between 5:30 a.m. and 10:30 a.m.). Based on this response, the first EMA survey will be scheduled at wake-up time plus 30 min. Subsequently, four fixed-time daily surveys will be administered: upon awakening, at 11:00 a.m., 3:00 p.m. and 8:30 p.m. Participants will be instructed to complete each survey immediately upon receiving the notification. Non-response will trigger up to three reminder notifications at 15 min intervals, after which the survey will expire and disappear.

Upon completion of the 14-day protocol, participants will return the actigraphy devices using a prepaid envelope provided by the study team, complete an end-of-study questionnaire assessing study acceptability and be invited to participate in a qualitative interview with a member of the research team.

### Outcomes

#### Primary outcomes

The primary outcomes are feasibility and acceptability.

**Feasibility** will be evaluated through: (1) attrition rates: measured by the number of participants who withdrew from the study, (2) EMA response rate: the number and percentage of data collected from EMA surveys relative to the number of potential assessments, as well as the latency between the prompt and participants' response, (3) actigraphy adherence (the threshold is ≥21 hours of wear time per 24 hours across the 14-day protocol).^27^

**Acceptability** will be assessed using a study-specific questionnaire and qualitative interviews. Quantitative indicators will include participant satisfaction, perceived relevance, ease of use and daily burden associated with EMA and actigraphy use.^27 28^ Responses will be rated on a five-point Likert scale (1=strongly disagree to 5=strongly agree). An item will be considered validated if the participant’s score is ≥3/5. Item-level acceptability will be defined as ≥70% agreement for positively worded items and ≤30% agreement for negatively worded items. During the interviews, the interviewer will take notes and, with the participant’s prior consent, audio-record the session. These recordings will be transcribed in a non-verbatim format and analysed using content analysis to identify recurring themes.

#### Secondary outcomes

Secondary outcomes will include the examination of immediate and lagged associations between pain, fatigue and sleep and objectively measured physical activity over the 14-day monitoring period, accounting for reciprocal relationships and variations across different times of the day. These analyses will describe both concurrent and delayed effects of these determinants on physical activity. In addition, adherence to and acceptability of two actigraphy devices worn at different body sites will be compared to evaluate their feasibility for assessing physical activity, sleep and sedentary behaviour.

### Sample size

This is a pilot study of feasibility and acceptability, without a quantitative primary outcome measure. As such, a formal sample size calculation is not required. In accordance with recommendations and considering what is reasonable and feasible, we estimate that recruiting 50 participants will provide sufficient variability to assess the feasibility and acceptability of the study.^29 30^ This sample size estimate accounts for an anticipated attrition rate of approximately 20%.

### Eligibility criteria

Adults (≥18 years) with a confirmed diagnosis of RA or SpA, established by a rheumatologist, are eligible for inclusion. Participants must be able to understand and consent to the study protocol, be affiliated with a social security system and have access to a digital device with internet access (eg, smartphone or tablet).

Non-inclusion criteria include: insufficient proficiency in the French language; legal incapacity or deprivation of liberty; major cognitive impairment incompatible with repeated questionnaire completion (eg, Alzheimer’s disease); pregnant or breastfeeding women, as well as individuals with a history of alcohol or substance abuse, or severe psychiatric disorders that could affect adherence to the protocol or the reliability of collected data, are also excluded.

### Recruitment and consent

Physicians and nursing staff in the rheumatology department will inform potential participants about the study and provide them with a flyer, an information sheet and a non-opposition form. Passive recruitment will also be implemented: posters describing the study will be displayed within the rheumatology department. These posters will include a QR code linking to a video created by the research team, which explains the study, how the mobile app works, its objectives and expected outcomes and benefits for patients. The video will be hosted on a dedicated website that also provides additional information about the study. Once the patient’s non-opposition form has been obtained, a research team member will schedule a remote onboarding session to (i) verify eligibility, (ii) assist with app installation and (iii) explain device use. Actigraphy devices will be mailed with a prepaid return envelope.

### Participant timeline and data collection methods

**Figure 1:**
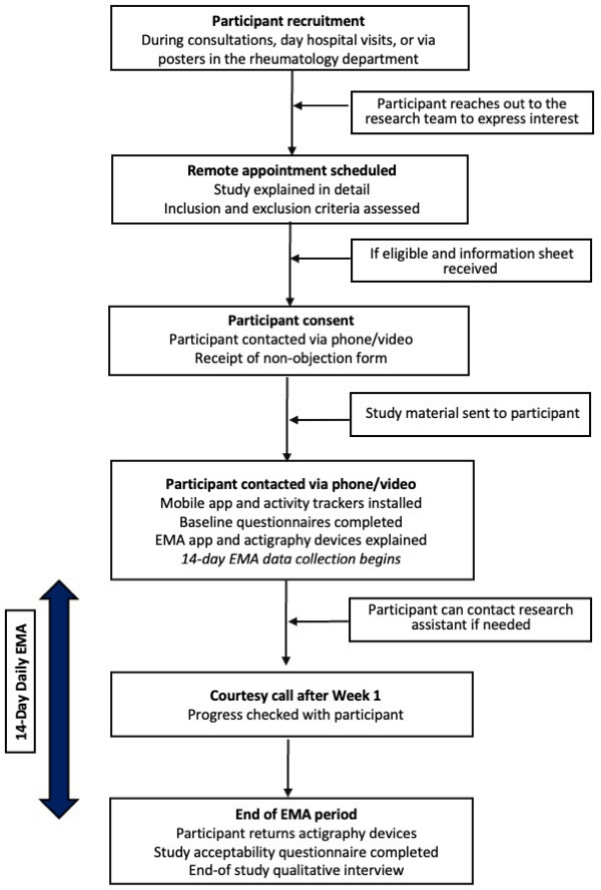
Study timeline. EMA, ecological momentary assessment.

The participant timeline is summarised in figure 1. The study started on 19 November 2025 and will continue until the target sample size of 50 participants is reached, which is expected to occur by the end of March 2026.

### Baseline assessment

At study baseline, participants will self-report the following data and questionnaires (for a full list of questionnaires and their description, see online supplemental file 1):

#### Clinical and sociodemographic data

Participants’ clinical characteristics include age, sex, weight, height and body mass index (BMI). Disease-specific variables comprise the type of chronic inflammatory rheumatism (spondyloarthritis or rheumatoid arthritis), disease duration (year of diagnosis), ongoing treatments and self-reported disease activity assessed with a visual analogue scale (VAS).^31^ In addition, sociodemographic information will be collected, encompassing employment status, educational attainment, household composition, living environment and housing type.

#### Questionnaires

Quality of life / physical activity / sleep: Health-related quality of life (EQ-5D-5L),^32^ Global Physical Activity Questionnaire (GPAQ),^33^ Pittsburgh Sleep Quality Index (PSQI),^34^ Inflammatory Arthritis Facilitators and Barriers (IFAB).^35^

Psychological factors: Motivation Scale towards Health-Oriented Physical Activity (EMAPS),^36^ Rosenberg Self-Esteem Scale (RSES),^37^ Pain Catastrophising Scale (PCS)^38^ and Hospital Anxiety and Depression scale (HADS-A).^39^

Fatigue and pain: Pichot Fatigue Scale,^40^ Fibromyalgia Rapid Screening Tool^41^ and pain measured using a VAS.

Disease activity / function (RA & SpA): Bath Ankylosing Spondylitis Disease Activity Index (BASDAI),^42 43^ auto-administered Ankylosing Spondylitis Disease Activity Score with C-Reactive Protein (ASDAS-CRP),^42 44^ auto-administered Disease Activity Score for 28 joints (DAS28)^45^ and the Routine Assessment of Patient Index Data 3 (RAPID3).^46 47^

#### Physical activity and sleep monitors

Participants will be mailed a study kit containing the two actigraphy devices detailed below.

The actigraph GT3X (Actigraph, Pensacola, FL, USA) is a compact device (5.1×4.1 × 1.5 cm) weighing 42 g, designed to record acceleration data as activity counts, providing an objective measure of body movement intensity. Participants will be instructed to wear the actigraph GT3X on their non-dominant wrist continuously for 14 days, except during water-based activities, such as bathing or swimming, during which the device must be removed.

The SENS device (SENS motion, SENS, Copenhagen, Denmark) is a lightweight actigraphy device weighing 20 g, worn on the front of the right thigh and secured with a hypoallergenic adhesive. Similar to the actigraph GT3X, it is worn continuously; however, it does not need to be removed during water-based activities.

#### EMA assessments

EMA details and results will be reported in alignment with an adapted STROBE checklist for reporting EMA studies.^48^ Participants will use the Avicenna app (https://avicennaresearch.com/), which is a General Data Protection Regulation-compliant research tool. To ensure that the items included in the EMA surveys will be relevant and appropriate for the target population, we conducted a simplified content validity procedure. First, items were selected based on a review of the literature on symptom assessment in RA and SpA and on existing validated questionnaires (eg, for pain, fatigue, mood and sleep). Draft items were then reviewed by a multidisciplinary research team (including experts in rheumatology, physical activity and sleep) as well as a patient representative to assess clarity, relevance and feasibility for repeated administration in daily life. Minor adjustments were made accordingly.

- Pain related to the disease will be assessed using the following item: *“Please select the number that best corresponds to the intensity of the pain you are experiencing right now”* (Numeric Rating Scale (NRS): 0=no pain, 10=extreme pain). Notifications will be delivered in the morning, at 11:00, 15:00 and 20:30.
- Fatigue will be assessed using the following item: *“Please select the number that best corresponds to your level of fatigue today”* (NRS: 0=no fatigue, 10=extreme fatigue). Notifications will be delivered in the morning, at 11:00, 15:00 and 20:30.
- Positive and negative affect will be assessed using items derived from the Circumplex Model of Affect.^49^ The two items *“How happy do you feel right now?”* (NRS: 0=not at all, 10=extremely happy) and *“How relaxed do you feel right now?”* (NRS: 0=not at all, 10=extremely relaxed) will be averaged to obtain a single score of positive affect. The two items *“How worried do you feel right now?”* (NRS: 0=not at all, 10=extremely worried) and *“How depressed do you feel right now?”* (NRS: 0=not at all, 10=extremely depressed) will be averaged to obtain a single score of negative affect. Notifications will be delivered in the morning, at 11:00, 15:00 and 20:30.
- Sleep quality will be assessed using the following item: *“How would you rate the quality of your sleep last night?”* (NRS: 0=poor, 10=excellent). This notification will be delivered only in the morning.

**Figure 2:**
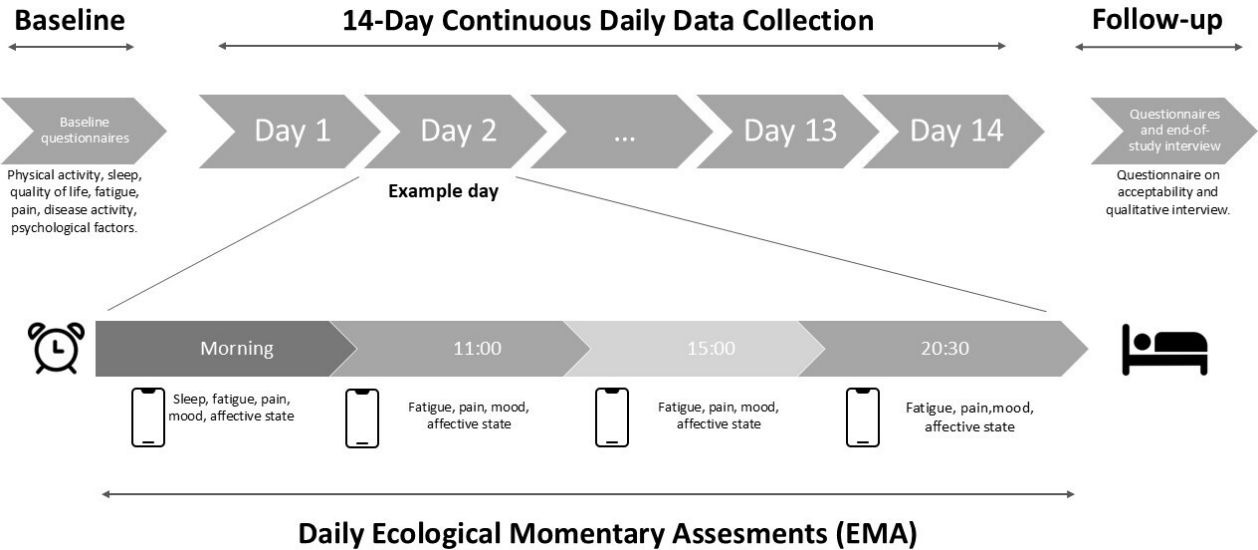
Study overview.

A full summary of the EMA questions is provided in text Box 1 and the study overview is presented in figure 2.

Box 1 **EMA surveys within the Avicenna app**

***Morning only***: How would you rate the quality of your sleep last night?

Poor — Excellent (0–10 slider).

Please select the score that best reflects the intensity of the pain you are feeling right now.

No pain — Extreme pain (0–10 slider).

Please select the score that best reflects your level of fatigue right now.

No fatigue — Extreme fatigue (0–10 slider).

How happy do you feel right now?

Not at all happy — Extremely happy (0–10 slider).

How relaxed do you feel right now?

Not at all relaxed — Extremely relaxed (0–10 slider).

How worried do you feel right now?

Not at all worried — Extremely worried (0–10 slider).

How depressed do you feel right now?

Not at all depressed — Extremely depressed (0–10 slider).

The application will be installed on participants’ smartphones after being downloaded from the App Store or Play Store. It will be used to send four short questionnaires per day to assess fatigue, sleep quality, positive and negative affect and pain, using a 0–10 scale. The Avicenna app will send audible alerts to participants’ smartphones at predefined intervals.

#### End of study assessments

Participants will complete the end-of-study questionnaire (online supplemental file 2) and participate in an end-of- study qualitative interview after the 14-day EMA collection period (online supplemental file 3).

Any adverse events relating to the use of technology or participating in the study will be recorded. Any serious adverse events will be reported to the sponsor.

### Statistical analysis

Analyses will be conducted once patient inclusion and follow-up are complete. Descriptive statistics will be reported as absolute frequencies and percentages for categorical variables, and as medians with IQRs for continuous variables. Where appropriate, means, SD and ranges (minimum and maximum) will also be provided. A flow diagram will summarise patient recruitment, exclusions and losses to follow-up, including reasons, and will define the final analysis population.

Feasibility and acceptability outcomes will be summarised for the overall study population using means, SD and range values (minimum and maximum). Boxplots will be used to visually depict data distributions. To assess participation rates, data will be dichotomised according to predefined thresholds, allowing estimation of the proportion of patients who declined participation. Reasons for lost to follow-up will be qualitatively analysed. Temporal trends will be described using time series, and response rates will be analysed over time intervals to highlight periods of high and low engagement.

For actigraphy, adherence to the 24-hour wear protocol over 14 days will be evaluated for both devices. Concordance between devices will be examined, and exploratory paired mean comparisons will be used to assess differences in wear time and perceived acceptability. Agreement between categorical acceptability ratings will be evaluated using Cohen’s Kappa coefficient.

### Patient and public involvement

A Patient Research Partner (PRP) living with RA was involved throughout the development of the study. This PRP contributed to refining study documents (protocol and patient information sheet) to ensure clarity and relevance for the target population and helped identify population-specific issues. In addition, the PRP participated in the simplified content validity process by reviewing and providing feedback on EMA items with respect to comprehensibility, relevance and response burden. Finally, the PRP also completed one full testing session of the EMA protocol to evaluate the feasibility of the procedures and identify potential practical issues.

### Potential implications

This study will evaluate the feasibility and acceptability of combining EMA and actigraphy to capture real-time interactions between fatigue, pain, mood, sleep and physical activity in patients with RA and SpA. Findings will guide optimisation of EMA protocols (eg, item selection, frequency, timing) and inform the design of a larger-scale study. The intensive nature of EMA may result in variable adherence or attrition; however, identifying these challenges is a core objective of this feasibility study and will directly inform protocol refinement for future trials.

### Ethics and dissemination

This study was approved by the French national ethics committee [Comité de protection des personnes Nord Ouest I] on 24/07/2025. Notification of the Committee’s approval was forwarded to the National Medicines Safety Agency (ANSM) by the sponsor (Grenoble Alpes University Hospital, Delegation for Clinical Research and Innovation).

### Dissemination of findings

Dissemination plans include presentations of the results at national and international conferences and publication in a peer-reviewed journal.

### Study status

Participant recruitment commenced on 19 November 2025.

## Supplementary material

10.1136/bmjopen-2025-113370 online supplemental file 1

10.1136/bmjopen-2025-113370 online supplemental file 2

10.1136/bmjopen-2025-113370 online supplemental file 3

## Data Availability

Data sharing not applicable as no datasets generated and/or analysed for this study.
