# Supplementary material for: Ecological momentary assessment of daily patient-reported outcomes and actigraphy-measured physical activity and sleep in patients with rheumatoid arthritis and spondyloarthritis: a study protocol
Source: BMJ Open. 2026 Feb 10;16(2):e113370. doi: 10.1136/bmjopen-2025-113370 (PMC12911789; doi:10.1136/bmjopen-2025-113370)
Supplement: online supplemental file 1 [file bmjopen-16-2-s001.pdf]

Full list and description of baseline questionnaires

Health-related quality of life (EQ-5D-5L): The EQ-5D-5L is a brief, multi-attribute, generic, health status measure composed of 5 questions with Likert response options (descriptive system) and a visual analog scale (EQ-VAS). The latter asks patients to rate their own health from 0 to 100 (the worst and best imaginable health, respectively). The descriptive system covers 5 dimensions of health (mobility, self-care, usual activities, pain or discomfort, and anxiety or depression) with 5 levels of severity in each dimension (no problems, slight problems, moderate problems, severe problems, and unable to perform or extreme problems) (29).

Global Physical Activity Questionnaire (GPAQ): The GPAQ is a questionnaire developed by the World Health Organization (WHO) to assess physical activity levels in diverse populations. It measures physical activity across three domains: Work-related activity (occupational or domestic tasks, Travel-related activity (walking or cycling for transportation), Leisure-time activity (exercise, sports, or recreational activities). The GPAQ also captures sedentary behavior and provides data on the frequency, intensity, and duration of activities (30).

Pittsburgh Sleep Quality Index (PSQI): The PSQI is a self-reported questionnaire that assesses sleep quality and quantity in terms of seven components, namely, subjective sleep quality, sleep latency, sleep duration, habitual sleep efficiency, sleep disturbances, use of sleeping medications, and daytime dysfunction (31).

Inflammatory Arthritis Facilitators and Barriers (IFAB) : The IFAB Questionnaire is designed to identify and document factors influencing engagement in physical activity among individuals with inflammatory Arthritis (IA). Items are grouped into four categories: psychological status, social support, disease-related factors, and environmental factors. The IFAB consists of 10 items, each rated on a scale from 0 to 10, where “0” indicates no impact and “10” represents the maximum impact on physical activity. Facilitators are scored positively, while barriers are scored negatively. The questionnaire has been validated specifically for patients with IA (32).

Motivation Scale towards Health-Oriented Physical Activity (EMAPS): The EMAPS is an 18-item scale designed to measure self-determined motivation toward physical activity. It measures 6 dimensions of motivation: intrinsic motivation, identified regulation , integrated regulation, introjected regulation, external regulation, and amotivation (33).

Rosenberg Self-Esteem Scale (RSES) : The RSES is a self-report instrument designed to assess an individual's self-esteem. It consists of 10 items rated on a 4-point Likert scale, ranging from "strongly agree" to "strongly disagree" (34).

Pain Catastrophizing Scale (PCS) : The PCS is a 13-item self-report instrument designed to assess catastrophic thinking related to actual or anticipated pain. It conceptualizes catastrophizing as a multidimensional construct, comprising three subscales: rumination, magnification, and helplessness (35).

Hospital Anxiety and Depression scale (HADS-A) : The HADS is designed to screen for clinically significant symptoms of anxiety and depression. The anxiety subscale (HADS-A) includes items assessing generalized anxiety features such as tension, worry, fear, panic, difficulty relaxing, and restlessness. Respondents rate their current feelings on a 4-point Likert scale, ranging from 0 to 3 (36).

Pichot Fatigue Scale : The Pichot Fatigue Scale is a brief self-report inventory consisting of 24 items. Factor analysis has identified three homogeneous subscales of eight items each, assessing depressive mood, asthenia/fatigue, and anxiety dimensions (37).

Fibromyalgia Rapid Screening Tool (FIRST) : It is a Patient Reported Outcome Measures consisting of 6 questions to which the patients answer "yes" or "no." Positive answers to 5 or more questions allow for FM diagnosis. In a development study12, the FiRST showed sensitivity of 90.5% and specificity of 85.7% (area under the curve [AUC] of 0.93) in diagnosing fibromyalgia (according to the 1990 ACR criteria), while also showing good consistency of the test over time (intraclass correlation coefficient, 0.87) (38).

Bath Ankylosing Spondylitis Disease Activity Index (BASDAI) : The BASDAI measures patient-reported disease activity in individuals with AS. First published in 1994, it originally used visual analog scales. The index captures self-reported levels of back pain, fatigue, peripheral joint pain and swelling, localized tenderness, and both the duration and severity of morning stiffness. Responses are recorded on either a numeric rating scale (0–10) or a visual analog scale (VAS, 0–10 cm), anchored by the descriptors "none" and "very severe." Morning stiffness duration is assessed using a time-based scale ranging from 0 to 2 or more hours (39, 40).

Ankylosing Spondylitis Disease Activity Score with C-Reactive Protein (ASDAS-CRP) : The ASDAS is designed to assess disease activity in AS using a composite index that combines patient-reported outcomes and objective measures. It incorporates clinically relevant domains

for both patients and clinicians. Initially introduced in 2008 in four draft versions, the tool was refined to two final validated versions, endorsed by the ASAS. The score includes patient-reported evaluations of back pain, morning stiffness duration, peripheral joint pain and/or swelling, and overall well-being, alongside an objective inflammatory marker (either erythrocyte sedimentation rate [ESR] or CRP) (39, 41).

Disease Activity Score for 28 joints (DAS28) : The DAS and its shortened version, DAS28, were developed to assess disease activity in RA in both clinical practice and clinical trials, at individual and group levels. DAS/DAS28 is a continuous measure that integrates data on swollen and tender joints, an acute phase reactant, and the patient's global assessment of health. The DAS-based EULAR response criteria, primarily intended for clinical trials, classify patients as non-, moderate-, or good responders based on both the degree of improvement and the level of disease activity achieved (42).

Routine Assessment of Patient Index Data 3 (RAPID3) : The RAPID3 is a self-administered tool developed to assess disease activity in patients with RA. It relies exclusively on PROs, without requiring joint examination or laboratory biomarkers. The questionnaire is sensitive to changes in disease activity and effectively differentiates between various levels of disease severity. Due to its simplicity and rapid completion time, approximately 1.5 minutes, RAPID3 is recommended by the American College of Rheumatology (ACR) for routine use in clinical practice (43, 44).
