# Supplementary material for: Ecological momentary assessment of daily patient-reported outcomes and actigraphy-measured physical activity and sleep in patients with rheumatoid arthritis and spondyloarthritis: a study protocol
Source: BMJ Open. 2026 Feb 10;16(2):e113370. doi: 10.1136/bmjopen-2025-113370 (PMC12911789; doi:10.1136/bmjopen-2025-113370)
Supplement: online supplemental file 2 [file bmjopen-16-2-s002.pdf]

**End-of-Study Questionnaire**

*Part 1 – Experience with the Daily Questionnaires (EMA)*

Please indicate to what extent you agree with each of the following statements:

Scale: 1 = Not at all | 2 = A little | 3 = Moderately | 4 = Quite a lot | 5 = Completely

1. I felt comfortable answering the daily questionnaires.
2. It was easy to answer the daily questionnaires.
3. Answering the daily questionnaires made me more aware of my physical activity level.
4. The daily questionnaires motivated me to be more physically active.
5. Answering the questionnaires helped me become more aware of my feelings.
6. I looked forward to answering the daily questionnaires.

*Part 2 – Response Frequency and Interference*

Please indicate how often you experienced the following situations:

Scale: 1 = Never | 2 = Rarely | 3 = Sometimes | 4 = Often | 5 = Always

7. I ignored or did not answer a daily questionnaire.
8. It was difficult to find the time to answer the questionnaires.
9. The questionnaires interfered with my daily routine.
10. There were many technical problems with the questionnaires.

*Part 3 – Experience with the Physical Activity Sensor*

Please indicate to what extent you agree with the following statements:

Scale: 1 = Not at all | 2 = A little | 3 = Moderately | 4 = Quite a lot | 5 = Completely

11. It was easy to wear the physical activity sensor on my wrist.
12. The sensor was comfortable to wear on my wrist.
13. Wearing the wrist sensor interfered with my daily routine.
14. It was easy to wear the physical activity sensor on my thigh.
15. The sensor on my thigh was comfortable to wear.
16. Wearing the thigh sensor interfered with my daily routine.

*Part 4 – Overall Acceptability of the Study*

Please indicate to what extent you agree with the following statements:

Scale: 1 = Definitely not | 2 = Probably not | 3 = Maybe | 4 = Probably yes | 5 = Definitely yes

17. I would recommend someone else to take part in a study like this.
18. I would be willing to participate in a similar study in the future.
