# Supplementary material for: Ecological momentary assessment of daily patient-reported outcomes and actigraphy-measured physical activity and sleep in patients with rheumatoid arthritis and spondyloarthritis: a study protocol
Source: BMJ Open. 2026 Feb 10;16(2):e113370. doi: 10.1136/bmjopen-2025-113370 (PMC12911789; doi:10.1136/bmjopen-2025-113370)
Supplement: online supplemental file 3 [file bmjopen-16-2-s003.pdf]

Thematic Guide for End-of-Protocol Qualitative Interviews

Technical issues
Notifications

- Did you experience any problems with the notifications?

Missed questionnaires

- Were there situations where you were not notified of a questionnaire even though you were near your phone and it was not completely in silent mode? How do you explain this?

Uncompleted questionnaires

- On what occasions were you unable to complete a questionnaire even though you were aware of the request? On average, how many times per day did this occur?

Constraints

- On what occasions did you find completing the questionnaire bothersome (unpleasant/uncomfortable/inappropriate)? How did you handle this?

Avicenna application

- Do you have any feedback or recommendations regarding the Avicenna application?

Questionnaires
Number of questionnaires

- Looking back, how do you perceive the number of questionnaires you had to complete each day? (If the participant hesitates, offer the following options: "I could have done more," "It was just right," or "It was too many.")

Length of questionnaires

- Looking back, how do you perceive the length of the questionnaires? (If the participant hesitates, offer the following options: "They could have been longer," "It was just right," or "They were too long.")
- Would you be willing to complete more questionnaires if each one contained fewer questions?
- Would you be willing to complete the same number of questionnaires if each contained more questions?
- Morning/evening questionnaires: Did the first questionnaire in the morning or the last in the evening disrupt your sleep schedule? What adjustments would you suggest?

Questions
Problematic questions

- Did any of the questions cause difficulties, for example due to lack of clarity, ambiguity, or missing response options? Which ones? How would you suggest modifying them?
    - Have a printed copy of the questionnaire available and, if necessary, allow the participant to refer to it.

Actigraphy
Daily wear

- Can you describe your daily experience wearing the actigraph monitors? (Ease, forgetfulness, discomfort, routine, etc.)
- Did you notice a difference in comfort or discomfort between the two actigraph monitors (thigh vs. wrist)? Which one, and in what situations?

Comfort and acceptability

- At certain times, did you feel any physical discomfort (chafing, weight, heat, visibility, etc.) related to either of the actigraph monitors?
- During which activities (sleep, sport, work, social life, etc.) did wearing one of the devices seem restrictive?
- Were you ever tempted to remove one of the actigraph monitors? If yes, under what circumstances?

Aesthetics and social perception

- Did the visible wearing of the actigraph monitors on your wrist or thigh generate reactions from others or from yourself? If yes, which ones?
- Did you feel embarrassed or self-conscious in public while wearing these devices?

Perceived reliability and understanding of the device

- Did you have an idea of what each sensor was measuring (wrist vs. thigh)? Did this influence how you wore them or how you perceived their usefulness?
- If you had to wear only one of the two devices, which would you choose? Why?

General
Study start

- How would you assess the information you received about the study? How would you assess the documents provided? Were there any pieces of information you felt were missing?

Technical issues and support

- Did you encounter technical difficulties during the study? Which ones?
- Did you receive adequate support/assistance?
- If technical support had been available, how many times would you have wanted to contact it?

Experience

- Overall, how would you rate your experience in this study on a scale of 1 to 10? (1 = very negative, I would not participate in a similar study again / 10 = very positive, I would participate again without hesitation).
