# Supplementary material for: Ecological momentary assessment of daily patient-reported outcomes and actigraphy-measured physical activity and sleep in patients with rheumatoid arthritis and spondyloarthritis: a study protocol
Source: BMJ Open. 2026 Feb 10;16(2):e113370. doi: 10.1136/bmjopen-2025-113370 (PMC12911789; doi:10.1136/bmjopen-2025-113370)
Supplement: Supplementary file 4 [file bmjopen-2025-113370.reviewer_comments.pdf]

PEER REVIEW HISTORY

BMJ Open publishes all reviews undertaken for accepted manuscripts. Reviewers are asked to complete a checklist review form and are provided with free text boxes to elaborate on their assessment. These free text comments are reproduced below.

ARTICLE DETAILS

Title (Provisional)

Ecological Momentary Assessment of Daily Patient Reported Outcomes and Actigraphy-Measured Physical Activity and Sleep in Patients with Rheumatoid Arthritis and Spondyloarthritis: A Study Protocol

Authors

aymard, nathan; Darmaillacq, Agathe; Bailly , Sebastien; Kechichian, Amélie; Baillieul, Sébastien; Bernardy, Chloé; GASTALDI, Romain; Flore, Patrice; Baillet, Athan; Mendelson, Monique

---

VERSION 1 - REVIEW

**Reviewer**	**1**
**Name**	**Gronhaug, Gudmund**
**Affiliation**	**østfold hospital trust, Department of physical medicine and rehabilitation**
**Date**	**09-Jan-2026**
**COI**	**None**

Thank you for a well written and interesting protocol.

I find the study relevant and the results from this study might give some insight to help find better treatment algorithms for Chronic inflammatory rheumatic diseases.

However I do have some minor concerns about the protocol that I hope the authors might take in consideration.

In both the abstract and the manuscript it is clearly stated that the study is designed to look at Chronic inflammatory rheumatic diseases. Still, the only two diagnoses mentioned is Rheumatoid arthritis and spondyloarthritis.

It leaves me questioning if the title and methods needs to be rewritten or if this is a slip by the authors.

I would suggest to rewrite the protocol to ensure that the methods and title are reflecting the study population that is intended to take part in the study; either all kinds of CIRDS or a specific sub population.

I would also recommend to rewrite the first two paragraphs of the introduction to further highlight why the study aims to monitor everyday activity of the participants.

Ecological momentary assessments do have potentially high drop outs based on the intervening design for the subjects taking part. With this in mind it seems to be risky to involve as few as 50 subjects with the need of a success rate of 80%.

If possible I would recommend to include at least 10 more participants to ensure that the study results is possible to use with a higher drop out rate.

VERSION 1 - AUTHOR RESPONSE

Reviewer 1

We thank the reviewer for the positive assessment of the relevance and quality of our protocol, as well as for the helpful suggestions.

Comment 1

I would suggest to rewrite the protocol to ensure that the methods and title are reflecting the study population that is intended to take part in the study; either all kinds of CIRDS or a specific sub population.

Response 1

We appreciate this observation. We agree that clarification was needed. We have revised the title, methods section, and relevant parts of the manuscript to clearly reflect that the study population includes patients with rheumatoid arthritis and spondyloarthritis specifically. This ensures consistency between the title, objectives, and study population.

"Ecological Momentary Assessment of Daily Patient Reported Outcomes and Actigraphy-Measured Sleep and Physical Activity in Patients with Rheumatoid Arthritis and Spondyloarthritis ~~Chronic Inflammatory Rheumatic Diseases~~: A Study Protocol"

Comment 2

I would also recommend to rewrite the first two paragraphs of the introduction to further highlight why the study aims to monitor everyday activity of the participants.

Response 2

We thank the reviewer for this suggestion. The first two paragraphs of the Introduction have been rewritten to better emphasize the rationale for monitoring participants' everyday activity, including its clinical relevance and its potential contribution to understanding disease burden in daily life. Here is the rewritten introduction (in **red**) as well as the initial introduction (in **black**) below.

"Patient-reported outcome measures (PROs), including pain, fatigue, mood, and sleep quality, are major determinants of impaired quality of life in individuals with rheumatoid arthritis (RA) and spondyloarthritis (SpA) (1, 2). These chronic conditions affect the joints and surrounding tissues and are characterised by symptoms that fluctuate markedly within and between days (3-6). Pain, fatigue, and sleep disturbances frequently co-occur and interact dynamically, influencing patients' daily functioning and levels of physical activity and sedentary behavior

(7, 8). As a result, the burden of RA and SpA is not only defined by average symptom levels, but also by their day-to-day variability and temporal interactions, which are insufficiently captured by traditional cross-sectional or clinic-based assessments.

In addition to pharmacological treatments such as nonsteroidal anti-inflammatory drugs (NSAIDs) and conventional or biological disease-modifying antirheumatic drugs (cs/bDMARDs), regular physical activity (PA) and reduced sedentary behaviour are strongly recommended for patients with RA and SpA (9-11). Current European League Against Rheumatism (EULAR) guidelines emphasise regular PA as a core component of disease management due to its beneficial effects on pain, physical function, mobility, and disease activity (12-16). However, despite these established benefits, many patients struggle to maintain regular PA in daily life, with pain, fatigue, and sleep disturbances commonly reported as major barriers (17-19). Understanding how fluctuations in symptoms relate to everyday activity patterns in real-world settings is therefore essential to identify periods of vulnerability and opportunity, and to inform more personalised and adaptive PA interventions."

"Patient-Reported Outcomes (PROMs)—such as pain, persistent fatigue, mood disturbances, and sleep problems—represent major determinants of impaired quality of life in individuals with Chronic Inflammatory Rheumatic Diseases (CIRDs). These disorders, which include rheumatoid arthritis (RA) and spondyloarthritis (SpA), are chronic conditions affecting the joints and surrounding tissues (1, 2). CIRDs are characterized by acute and chronic pain, frequently involving peripheral joints and/or the spine, and may result in progressive joint damage as well as systemic complications (3-5).

In addition to pharmacological treatments such as nonsteroidal anti-inflammatory drugs (NSAIDs) and conventional or biological disease-modifying antirheumatic drugs (cs/bDMARDs), regular physical activity (PA) and reduced sedentary behavior are strongly recommended patients with CIRDs (9-11). In its guidelines on lifestyle and dietary measures for CIRDs, EULAR (European League Against Rheumatism) recommends regular PA for patients with SpA and RA (15, 16). Regular PA is associated with reduced pain, improved mobility and physical function (14, 15), as well as decreased disease activity (12-16). Yet, despite these benefits, many patients report difficulties maintaining regular PA, with pain, fatigue, and sleep disturbances being major barriers (17-19)."

Comment 3

Ecological momentary assessments do have potentially high drop outs based on the intervening design for the subjects taking part. With this in mind it seems to be risky to involve as few as 50 subjects with the need of a success rate of 80%. If possible I would recommend to include at least 10 more participants to ensure that the study results is possible to use with a higher drop out rate.

Response 3

We thank the reviewer for raising the important issue of potential dropout and adherence in EMA studies. We fully agree that participant burden is a critical consideration when designing intensive longitudinal protocols.

Existing evidence supports the feasibility of EMA protocols across diverse populations, including those potentially facing higher burden or health-related constraints. A recent

systematic review of EMA studies in movement behavior research among adolescents and emerging adults reported overall compliance rates ranging from approximately 77% to over 90%, depending on prompting frequency, with study duration not being a major determinant of compliance (Wang et al., 2025). Similarly, a systematic review and meta-analysis in elderly populations reported a pooled EMA compliance rate of 86.4%, despite heterogeneity in protocols and health status (Yao et al., 2023). EMA feasibility has also been demonstrated in clinical populations such as people living with cancer, where response rates were generally acceptable and no systematic feasibility issues were reported (Kampshoff et al., 2019).

Taken together, these findings suggest that well-designed EMA protocols can achieve high compliance even in populations with increased vulnerability or symptom burden. On this basis, and given the exploratory and feasibility-oriented nature of the present study, we believe that the planned sample size remains appropriate. Rather than increasing the number of participants, this study aims to generate empirical data on adherence and acceptability that will directly inform sample size calculations and protocol optimisation for future larger-scale studies in people living with RA and SpA.

We added the following sentence to the "*Potential implications*" (page 13):

"The intensive nature of EMA may result in variable adherence or attrition; however, identifying these challenges is a core objective of this feasibility study and will directly inform protocol refinement for future trials."

References

- Wang S, Yang CH, Brown D, Cheng A, Kwan MYW. Participant Compliance With Ecological Momentary Assessment in Movement Behavior Research Among Adolescents and Emerging Adults: Systematic Review JMIR Mhealth Uhealth 2025;13:e52887 doi: 10.2196/52887
- Yao L, Yang Y, Wang Z, Pan X, Xu L. Compliance with ecological momentary assessment programmes in the elderly: a systematic review and meta-analysis. BMJ Open. 2023 Jul 12;13(7):e069523. doi: 10.1136/bmjopen-2022-069523. PMID: 37438069; PMCID: PMC10347474.
- Kampshoff CS, Verdonck-de Leeuw IM, van Oijen MG, Sprangers MA, Buffart LM. Ecological momentary assessments among patients with cancer: A scoping review. Eur J Cancer Care (Engl). 2019 May;28(3):e13095. doi: 10.1111/ecc.13095. Epub 2019 May 14. PMID: 31090160; PMCID: PMC9285429.
